# Anti-IL-20 monoclonal antibody inhibited inflammation and protected against cartilage destruction in murine models of osteoarthritis

**DOI:** 10.1371/journal.pone.0175802

**Published:** 2017-04-20

**Authors:** Yu-Hsiang Hsu, Ya-Yu Yang, Man-Hsiang Huwang, Yun-Han Weng, I-Ming Jou, Po-Tin Wu, Tain-Yu Lin, Li-Wha Wu, Ming-Shi Chang

**Affiliations:** 1 Institute of Clinical Medicine, College of Medicine, National Cheng Kung University, Tainan, Taiwan; 2 Research Center of Clinical Medicine, National Cheng Kung University Hospital, Tainan, Taiwan; 3 Research Center of New Antibody Drug, National Cheng Kung University, Tainan, Taiwan; 4 Department of Biochemistry and Molecular Biology, College of Medicine, National Cheng Kung University, Tainan, Taiwan; 5 Department of Orthopedics, National Cheng Kung University Hospital, Tainan, Taiwan; 6 Institute of Molecular Medicine, College of Medicine, National Cheng Kung University, Tainan, Taiwan; University of Alabama at Birmingham, UNITED STATES

## Abstract

Osteoarthritis (OA) is a degenerative joint disease characterized by progressive destruction of articular cartilage. Interleukin (IL)-20 is a proinflammatory cytokine involved in the pathogenesis of rheumatoid arthritis. We investigated the role of IL-20 in OA and evaluated whether anti-IL-20 antibody (7E) treatment attenuates disease severity in murine models of surgery-induced OA. Immunohistochemical staining was used to detect IL-20 and its receptors expression in synovial tissue and cartilage from OA patients, and in OA synovial fibroblasts (OASFs) and chondrocytes (OACCs) from rodents with surgery-induced OA. RTQ-PCR and western blotting were used to determine IL-20-regulated OA-associated gene expression in OASFs and OACCs. OA rats and OA mice were treated with 7E. Arthritis severity was determined based on the degree of cartilage damage and the arthritis severity score. We found that IL-20 and its receptors were expressed in OASFs and OACCs. IL-20 induced TNF-α, IL-1β, MMP-1, and MMP-13 expression by activating ERK-1/2 and JNK signals in OASFs. IL-20 not only upregulated MCP-1, IL-6, MMP-1, and MMP-13 expression, but also downregulated aggrecan, type 2 collagen, TGF-β, and BMP-2 expression in OACCs. Arthritis severity was significantly lower in 7E-treated OA rats, and 7E- or MSC-treated OA mice. Therefore, we concluded that IL-20 was involved in the progression and development of OA through inducing proinflammatory cytokines and OA-associated gene expression in OASFs and OACCs. 7E reduced the severity of arthritis in murine models of surgery-induced OA. Our findings provide evidence that IL-20 is a novel target and that 7E is a potential therapeutic agent for OA.

## Introduction

Osteoarthritis (OA), a slow progressing disease, causes articular cartilage fibrillation and loss. The articular cartilage is altered to some degree in all joints with OA. In addition to developing cartilage changes with aging, cartilage degeneration might occur in response to unsuitable mechanical stress and systemic or local low-level inflammation associated with trauma and obesity, which are critical risk factors for the development and progression of OA [1–3]. Although the destruction of articular cartilage is a major characteristic of OA, other joint tissue, including the synovial membrane and subchondral bone, also participate in the disease progression [4]. In the late stages of OA patients, the lack of disease-modifying OA drugs results in progressive cartilage damage. Therefore, surgical interventions are often necessary to partially recover joint function.

Interleukin (IL)-1β and tumor necrosis factor (TNF)-α released during synovitis, target on chondrocytes and suppress the production of type II collagen and aggrecan, the critical components of cartilage matrix [5, 6]. These proinflammatory cytokines promote the secretion of aggrecanase and matrix metalloproteinases, enzymes that degrade the matrices, causing cartilage damage [7, 8], and interfere the metabolic equilibrium of the cartilage matrix [9]. The cellular sources of these inflammatory cytokines and enzymes are not just the synovial cells but also the chondrocytes themselves, which contribute to cartilage damage and loss [10, 11].

IL-20, a member of IL-10 family (consisting of IL-10, IL-19, IL-20, IL-22, IL-24, and IL-26) has two receptor complexes: IL-20R1/IL-20R2 and IL-22R1/IL-20R2 [12]. IL-20 acts on synovial fibroblasts (SFs), endothelial cells, keratinocytes, and renal epithelial cells [12–14]. IL-20 is a proinflammatory cytokine that causes inflammation, angiogenesis, chemotaxis, and apoptosis, and is involved in the pathogenesis of psoriasis, atherosclerosis, stroke, rheumatoid arthritis (RA), acute and chronic renal failure, and prostate cancer [12–21].

Animal models of OA are used to study the pathogenesis of cartilage destruction and evaluate the therapeutic potential for treating OA. Surgically induced joint destabilization is the most widely used method for inducing OA in animals. The common two methods for surgical induction are: anterior cruciate ligament transection (ACLT) and destabilization of the medial meniscus (DMM) [22, 23]. These models allow the temporal control of disease induction and follow predictable progression of the disease. Surgically induced destabilization models of OA also show the similar molecular pathology and histopathology that is observed in OA patients [24]. Advantages of surgical models over spontaneous models include a faster onset of disease, less variability, and less dependence on genetic background [25].

Mesenchymal stem cells (MSCs) are multipotent stromal cells, which are a subset of nonhematopoietic adult stem cells that originate in the mesoderm. They are self-renewing and able to differentiate not only into mesoderm lineages, such as chondrocytes, osteocytes, and adipocytes, but also into ectodermic and endodermic cells. They can easily be isolated from the bone marrow, adipose tissue, the umbilical cord, fetal liver, muscle, and lung, and can be successfully expanded *in vitro* [26]. OA is considered a cartilage degenerative disease. Because of the limitations of chondrocyte regeneration and the irreversible destruction of cartilage, MSCs have recently been used in a clinical trail [27], which reported that the size of cartilage defect decreased while the volume of cartilage increased in the medial femoral and tibial condyles of the high-dose group.

We previously [14] showed that IL-20 not only induced RA synovial fibroblasts (RASFs) to produce monocyte chemoattractant protein-1, IL-6, and IL-8, but that it also enhanced chemotaxis of neutrophil. Moreover, anti-IL-20 monoclonal antibody (7E) treatment or electroporating soluble IL-20R1 plasmid DNA into rats with collagen-induced arthritis (CIA) reduced the arthritis severity, which suggested that IL-20 could be the therapeutic target for RA treatment [14, 15, 17]. However, little is known about the function of IL-20 in the pathogenesis of OA. In this study, we investigated the role of IL-20 in OA and whether 7E treatment attenuates disease severity in murine models of surgery-induced OA. We also examined and compared the therapeutic effects between 7E and MSCs in a mouse OA model.

## Materials and methods

### OA patients

We collected synovial tissues and cartilage samples from 8 patients with OA during knee joint replacement surgery. Written informed consent was obtained. The Ethics Committee of National Cheng Kung University Hospital approved the study. The methods were carried out in accordance with the approved guidelines. The human study was approved by the Human Research Review Committee of National Cheng Kung University Hospital.

### Isolation and culture of synovial fibroblasts and chondrocytes

Freshly isolated synovial tissue and cartilage obtained from OA rats was finely minced into 2- to 3-mm pieces and then digested for 1 hour at 37°C with 2 mg/ ml of Type Π Collagenase in Dulbecco’s modified Eagle’s medium (DMEM; Gibco BRL, Grand Island, NY). OA synovial fibroblasts (OASFs) and OA chondrocytes (OACCs) were separately cultured in DMEM containing 10% fetal bovine serum (FBS). All *in vitro* experiments used primary cultures of OASFs between passages 5 and 10.

### Immunohistochemical staining

The paraffin on the synovial tissues and cartilages were removed using xylene, and membranes were rehydrated using ethanol. The staining procedure was described in detail previously [14]. We used 3 μg/ml as the working concentration for each primary antibody and for the control mouse IgG1.

### Immunocytochemical staining

The OASFs and OACCs were grown on sterile glass slides (chamber slides; Nalge Nunc International, Rochester, NY), which were fixed in 3.7% paraformaldehyde and permeabilized using PBS with 0.1% Triton X-100. Nonspecific binding sites were blocked by incubating the OASFs and OACCs with background reducing components (DakoCytomation, Carpinteria, CA), then with primary antibodies (IL-20, IL-20R1, IL-20R2, or IL-22R1 monoclonal antibody), and then with secondary antibody using the same procedure as for immunohistochemical staining.

### Real-time quantitative (RTQ)-PCR

OASFs were plated for 1 day in DMEM with 10% FBS. The cells were incubated for 4 and 8 hours with 200 ng/ml of IL-20 to analyze TNF-α, IL-1β, matrix metalloproteinase (MMP)-1, and MMP-13 expression. OACCs were plated for 1 day in DMEM with 10% FBS and gene expression was analyzed. The cells were incubated for 6–8 hours with 200 ng/ml of IL-20 to analyze MMP-1, MMP-13, TGF-β, BMP-2, MCP-1, IL-6, aggrecan, and type 2 collagen (Col 2) expression. Total RNA was isolated (Invitrogen, Carlsbad, CA). Reverse transcription was done using reverse transcriptase (Clontech, BD Biosciences, Palo Alto, CA). TNF-α, IL-1β, MMP-1, MMP-13, TGF-β, BMP-2, MCP-1, IL-6, aggrecan, and Col 2 expression was then amplified on a thermocycler (LC 480; Roche Diagnostics, Roche Applied Science, Indianapolis, IN), with SYBR Green (Roche Diagnostics) as the interaction agent. Quantification analysis of messenger RNA (mRNA) was normalized with β-actin, which was used as the housekeeping gene. Relative multiples of changes in mRNA expression were determined by calculating 2^–ΔΔCt^.

### MMP activity

General MMP activity was measured using the Enzolyte 520 Generic MMP assay kit (AnaSpec, San Jose, CA). In brief, OASFs and OACCs were stimulated with IL-20 (200 ng/ml) for 24 and 48 hours and supernatants were harvested, centrifuged and incubated with p-aminophenylmercuric acetate for 3 hours at 37°C to activate MMPs. Aliquots of supernatants were then transferred to a 96-well plate and after addition of the 5-FAM (5-carboxyfluorescein) peptide substrate, fluorescence was measured at 490 nm (excitation)/520 nm (emission).

### Western blotting

OASFs were seeded at 1X10^6^ cells/dish and serum-starved for 4 hours before the cells were stimulated. OASFs were stimulated with 200 ng/ml of IL-20 for the time courses indicated below. Western blot analysis was done using antibody-specific phosphorylated ERK-1/2 and phosphorylated JNK (Cell Signaling Technology, Beverly, MA). Chondrogenic aggregates were analyzed using cell aggregates homogenized in lysis buffer after they had been treated with IL-20 for 7 days. The medium was replaced every 2 days, and aggregates were collected at the indicated time points for analysis. Protein concentrations were determined using a colorimetric kit (BCA protein assay; Thermofisher Scientific, Pierce, Rockford, IL) and an equal amount of protein was loaded in each experiment. Immunoblotting was done using antibody against phospho-Sox9 (ab59252; Abcam Plc, Cambridge, UK). β-actin (Cell Signaling Technology) was used as loading controls. Quantification of proteins in western bands was done using ImageJ software (http://imagej.nih.gov/ij/).

### Chondrogenesis and cell treatment

Mouse MSCs at subconfluent conditions were trypsinized, and aliquots of 2.5 × 10^5^ cells/well were added to a V-bottom polypropylene 96-well plate (Nunc, Thermo Scientific), and the plate was spun for 5 mins at 500 g. For differentiation into chondrocytes, cells were cultured in DMEM/HC (Caisson Labs, Smithfield, UT), 10^-7^M dexamethasone (Sigma-Aldrich, St. Louis, MO), 1μM ascorbate-2-phosphate (J.T.Baker, Avantor Performance Materials Taiwan Co. Ltd., Chu-Bei City, HsinChu Hsien), 1% sodium pyruvate (Caisson), ITS + Premix Tissue Culture Supplement (Becton Dickinson) and 10 ng/ml transforming growth factor (TGF)-β3 (Peprotech, Rocky Hill, NJ). The cell pellets formed free-floating aggregates within the first 24 hours. To analyze the effects of inflammatory cytokines, recombinant mouse IL-20 was added to the culture medium during chondrogenic induction. The medium was replaced every 3 days, and aggregates were collected at the indicated time points for analysis.

### Gene expression analysis in chondrogenesis

Total RNA was extracted from aggregates. Reverse transcription was done using reverse transcriptase (Invivogen, San Diego, CA). The expression of Sox 9 and type I collagen (Col 1) were then amplified on a thermocycler (LC 480; Roche Diagnostics), with SYBR Green (Roche Diagnostics) as the interaction agent. Quantification analysis of messenger RNA (mRNA) was normalized with β-actin, which was used as the housekeeping gene. Relative multiples of changes in mRNA expression were determined by calculating 2^–ΔΔCt^.

### OA rat model and treatment

All animal experiments were done according to the protocols based on the Taiwan National Institutes of Health (Taipei) standards and guidelines for the care and use of experimental animals. The research procedures were approved by the Animal Ethics Committee of National Cheng Kung University. The methods were carried out in accordance with the approved guidelines. Eight-week-old male Sprague-Dawley rats were anesthetized with ketamine and xylazine. The surgical procedure was described in detail previously [28]. After these procedures, the medial meniscus was completely removed. The meniscus was freed from both the medial and lateral attachments; the researcher tried to avoid damaging the underlying tibial cartilage. The joint capsule and skin were sutured with Vicryl and mononylon threads, respectively. The rats were divided into three groups of 5 rats each: OA controls, OA rats treated with mIgG (3 mg/kg) every 3 days, and OA rats treated 7E (3 mg/kg) every 3 days. All the rats were given an overdose of pentobarbital 8 weeks after the treatments had begun. Their knees were analyzed *in vivo* on a micro-CT (1076; Bruker MicroCT, Kontich, Belgium) with a high-resolution low-dose X-ray scanner. The knees were collected, cleaned, and then sectioned for toluidine blue staining.

### OA mouse model and treatment

C57BL/6 mice (8–10 weeks old) were obtained from the Laboratory Animal Center of National Cheng Kung University. OA was induced using DMM surgery on the right hind limb knee joint as previously described [22, 25]. The mice were divided into four groups (n = 5/group): OA controls, OA mice given an intra-articular injection of mIgG (2 μg/mouse 3 times a week), OA mice given an intra-articular injection of 7E (2 μg/mouse 3 times a week), and OA mice given an intra-articular injection of 5x10^5^ MSCs (single treatment) 14 days after surgery. Mice in the sham group was not dissected the medial meniscus ligament. The knee joints were collected for analysis 56 days after surgery.

### Histologic analysis of OA progression

Mouse knee joints were dissected and fixed in 4% paraformaldehyde. After they had been fixed, the joints were decalcified in 8% hydrochloric acid/formic acid working solution in phosphate buffered saline (PBS) and processed for histologic analysis. Paraffin-embedded knee joints were cut into 6-μm long sections that were then mounted on glass slides and stained with Safranin O and counterstained with Fast Green to analyze cartilage damage [29]. The scoring system [30] is recommended to apply to all four quadrants of the joint: medial femoral condyle (MFC), medial tibial plateau (MTP), lateral femoral condyle (LFC), and lateral tibial plateau (LTP). A score of 0 = normal cartilage, 0.5 = loss of PG with an intact surface, 1 = superficial fibrillation without loss of cartilage, 2 = vertical clefts and loss of surface lamina (any % or joint surface area), 3 = vertical clefts or erosion to the calcified layer lesion for 1–25% of the quadrant width, 4 = lesion reaches the calcified cartilage for 25–50% of the quadrant width, 5 = lesion reaches the calcified cartilage for 50–75% of the quadrant width, 6 = lesion reaches the calcified cartilage for >75% of the quadrant width.

### Statistical analysis

Prism 6.0 (GraphPad Software, San Diego, CA) was also used for the statistical analysis. A one-way analysis of variance (ANOVA) nonparametric Kruskal-Wallis test was used to compare the data between groups. Post hoc comparisons were done using Dunn's multiple comparison test. Significance was set at P < 0.05.

## Results

### Expression of IL-20 and its receptors in OA patients, OASFs, and OACCs

**Fig 1:**
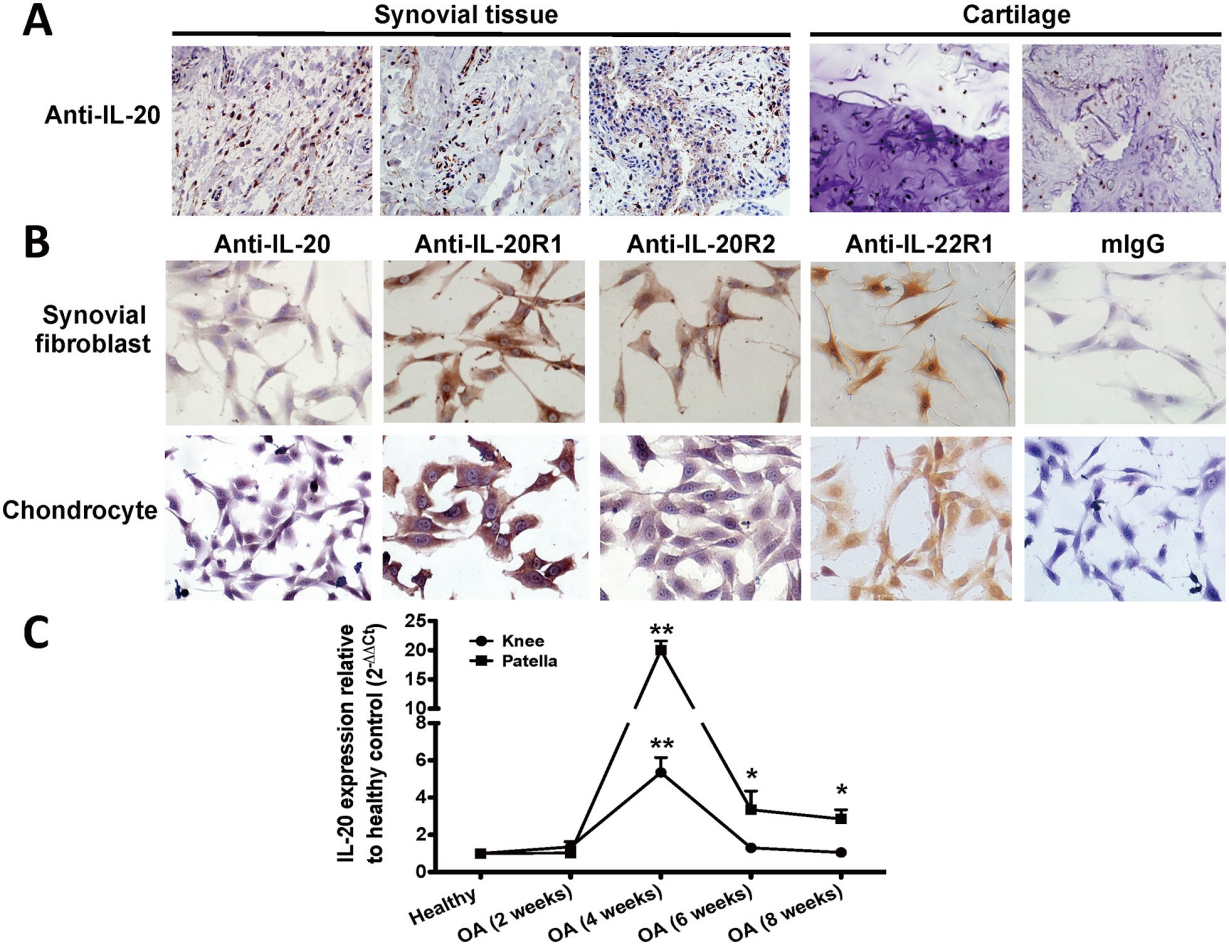
Expression of IL-20 and its receptors in patients with OA and in OA rats. (A) Interleukin (IL)-20 expression in osteoarthritis (OA) synovial tissue and cartilage was detected using immunohistochemical (IHC) staining with anti-IL-20 monoclonal antibody (7E). Staining with mouse immunoglobin G1 (mIgG1) isotype as the primary antibody was the negative control for IL-20. (B) IL-20, IL-20R1, IL-20R2, and IL-22R1 expression in OA synovial fibroblasts (OASFs) and OA chondrocytes (OACCs) isolated from OA rats were detected using anti-IL-20, anti-IL-20R1, anti-IL-20R2, and anti-IL-22R1 monoclonal antibodies. Staining with mIgG1 isotype as the primary antibody was the negative control for IL-20, IL-20R1, IL-20R2, and IL-22R1. The reaction was detected using AEC chromogen stain (red), and the nuclei were counterstained with hematoxylin (blue). Magnification was 200×. (C) The knees and patellae from OA rats at the indicated time periods were collected, mRNA was isolated, and the IL-20 transcript was measured using real-time quantitative polymerase chain reaction (RTQ-PCR). β-actin was an internal control. Data are the mean ± standard deviation (SD) of 5 mice. Data are representative of three independent experiments. *P < 0.05, **P < 0.01 compared with week 0.

To examine the role of IL-20 in the pathogenesis of OA, we first explored the expression of IL-20 and its receptors (IL-20R1, IL-20R2, and IL-22R1) in the synovial tissue and cartilage of patients with OA. Immunohistochemical analysis using antibodies against IL-20, IL-20R1, IL-20R2, and IL-22R1 showed that IL-20 and its receptors were all expressed in OA synovial tissue and cartilage (Fig 1A and S1A Fig). The mouse IgG1 isotype antibody used as a negative control showed no detectable immunoreactivity. In addition, we used PCR analysis to confirm the transcripts of IL-20 and its receptors in synovial tissue and cartilage isolated from patients with OA and had similar results (S1B Fig). To analyze the expression of IL-20 and its receptors in OASFs and OACCs, we used immunocytochemical staining on cells isolated from the synovial membranes and cartilage of OA rats. IL-20, IL-20R1, IL-20R2, and IL-22R1 were all expressed in rat OASFs and OACCs (Fig 1B). Mouse IgG1 isotype antibody was used as a negative control and showed no immunoreactivity. To analyze whether IL-20 was upregulated during the OA progression, we generated surgery-induced OA mice and compared with healthy mice. RTQ-PCR showed that IL-20 was significantly upregulated in the knees and patellae isolated from mice during OA development (Fig 1C).

### IL-20 induced TNF-α, IL-1β, MMP-1, and MMP-13 through ERK1/2 and JNK in OASFs

**Fig 2:**
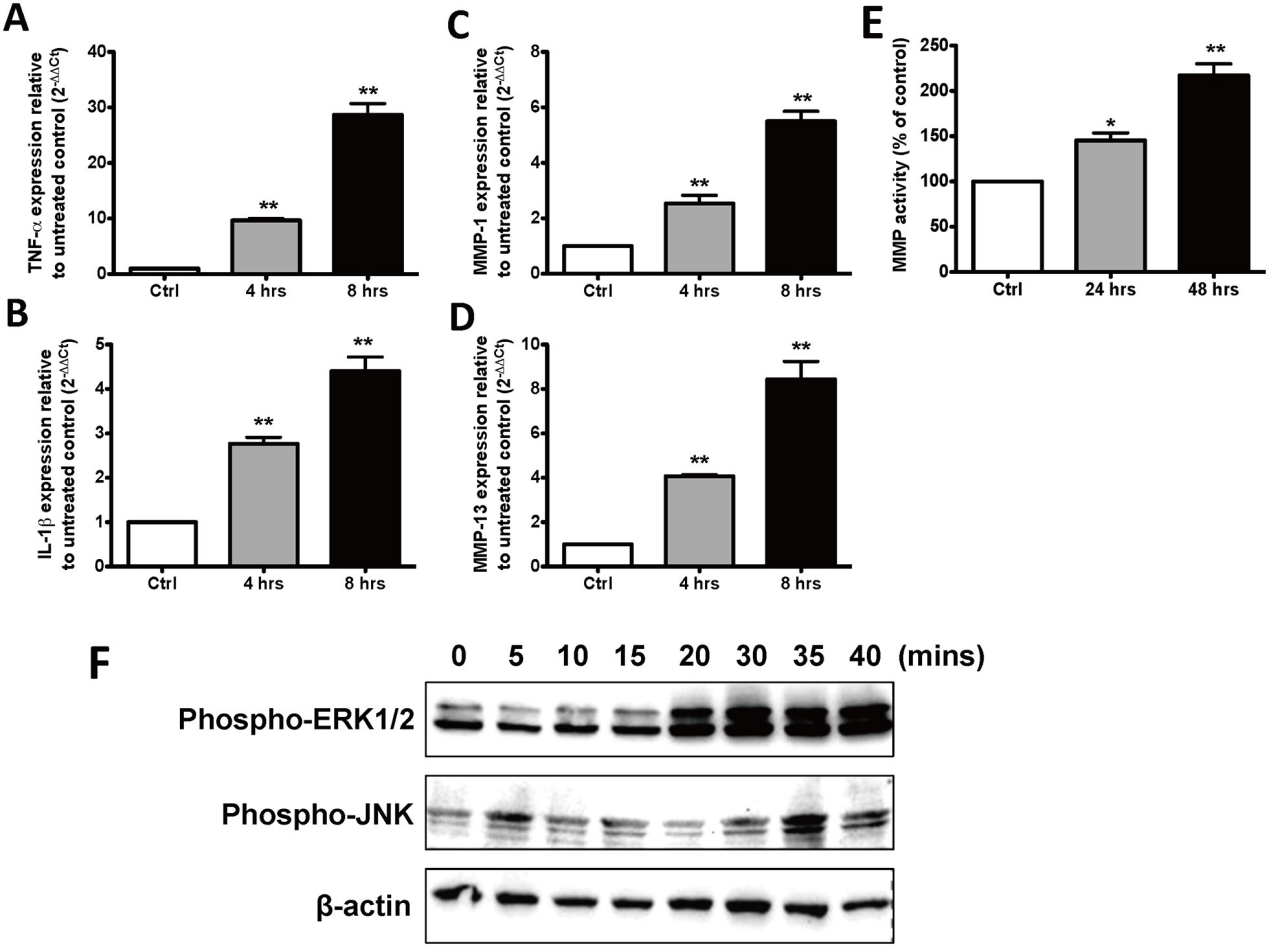
Functions of IL-20 in OASFs. (A-D) OASFs were treated with IL-20 (200 ng/ml) for 4 and 8 hours. mRNA was isolated and the transcripts of tumor necrosis factor (TNF)-α, IL-1β, matrix metalloproteinase (MMP)-1, and MMP-13 were analyzed using RTQ-PCR with specific primers. The quantification analysis of mRNA was normalized; β-actin was the housekeeping gene. *P < 0.05, **P < 0.01 versus untreated controls. Data are representative of three independent experiments, each done in triplicate. (E) MMP activity in the conditioned media of OASFs treated with IL-20 (200 ng/ml) for 24 and 48 hours. Results are shown as a percentage of the untreated control. *P < 0.05, **P < 0.01 versus untreated controls. Data are representative of three independent experiments, each done in triplicate. (F) OASFs were incubated with IL-20 (200 ng/ml) for the indicated time periods. Total cell lysates were analyzed using Western blotting with specific antibodies against phosphor-ERK-1/2, JNK, and β-actin. Data are representative of three independent experiments.

Among the proinflammatory cytokines involved in OA, TNF-α and IL-1β are considered the most important [4]. The pivotal proteinase that marks OA progression is MMP-13, the major type II collagen-degrading collagenase, which is regulated both by stress and by inflammatory signals [3]. We investigated whether IL-20 alters TNF-α, IL-1β, MMP-1, and MMP-13 expression *in vitro*. Therefore, we isolated and primary cultured SFs (OASFs) from OA rats to determine whether IL-20 upregulated the gene expression of these cytokines and proteinase. RTQ-PCR showed that IL-20 (200 ng/ml) induced TNF-α, IL-1β, MMP-1, and MMP-13 expression in OASFs (Fig 2A–2D). We also found that IL-20 increased MMP activity in OASFs (Fig 2E). We previously reported [14] that IL-20 activated the ERK-1/2 signaling in RA synovial fibroblasts (RASFs). Thus, we wanted to analyze whether IL-20 activates the same signals in OASFs. OASFs were treated with IL-20, and cell lysate was used for western blotting with an antibody specifically against phosphorylated ERK-1/2 and JNK. Phosphorylated ERK-1/2 and JNK expression increased after the OASFs had been stimulated with 200 ng/ml of IL-20, and the expression level was time-dependent (Fig 2F).

### L-20 upregulated IL-6, MCP-1, MMP-1, and MMP-13 in OACCs

**Fig 3:**
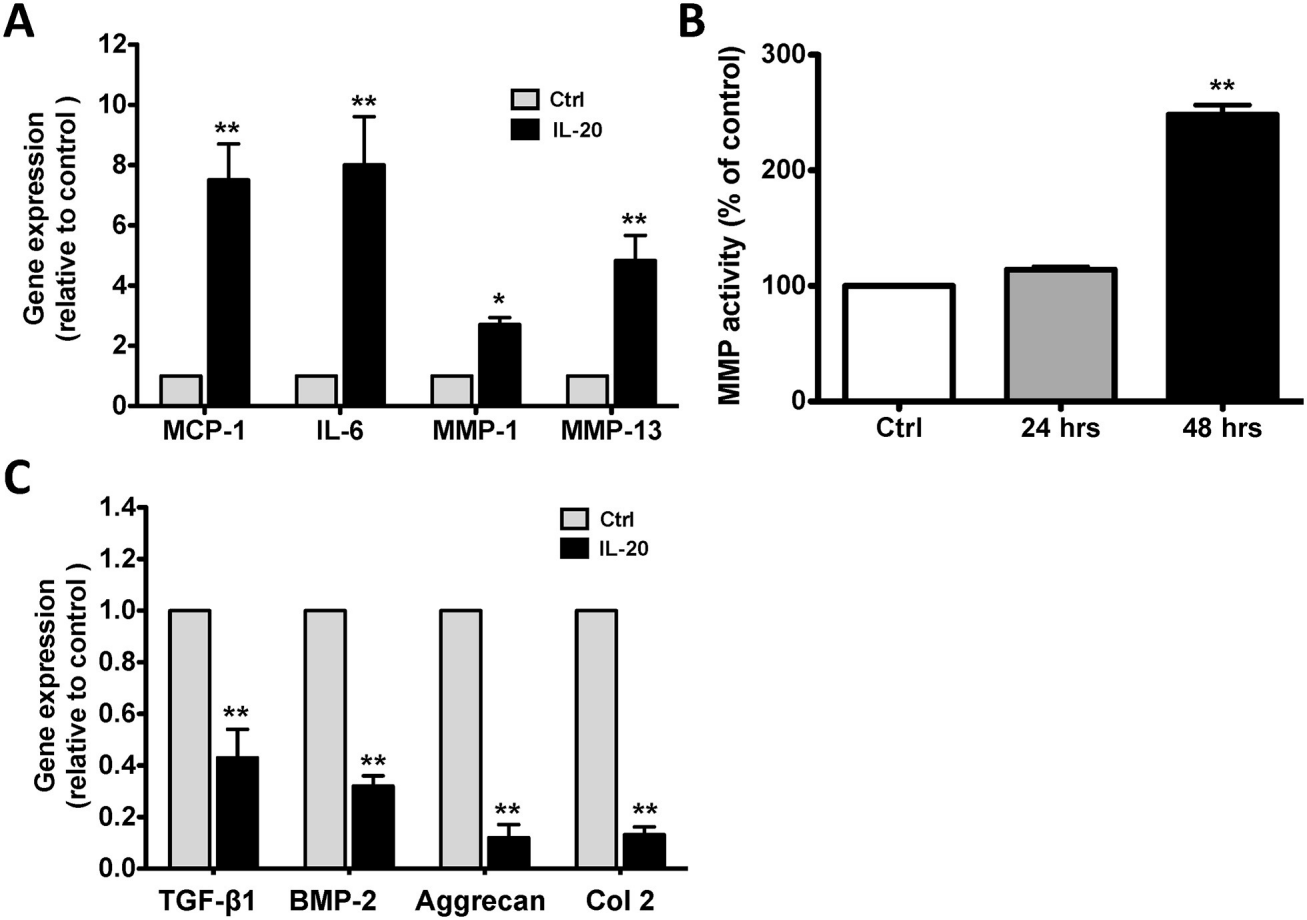
Functions of IL-20 in OACCs. (A) OACCs were treated with IL-20 (200 ng/ml) for 8 hours. mRNA was isolated and the transcripts of monocyte chemoattractant protein (MCP)-1, IL-6, MMP-1, and MMP-13 were analyzed using RTQ-PCR with specific primers. The quantification analysis of mRNA was normalized; β-actin was the housekeeping gene. *P < 0.05, **P < 0.01 versus untreated controls. Data are representative of three independent experiments, each done in triplicate. (B) MMP activity in the conditioned media of OACCs treated with IL-20 (200 ng/ml) for 24 and 48 hours. Results are shown as a percentage of the untreated control. **P < 0.01 versus untreated controls. Data are representative of three independent experiments, each done in triplicate. (C) OACCs were treated with IL-20 (200 ng/ml) for 6 hours. mRNA was isolated and the transcripts of TGF-β1, BMP-2, aggrecan, and type 2 collagen (Col 2) were analyzed using RTQ-PCR with specific primers. The quantification analysis of mRNA was normalized; β-actin was the housekeeping gene. **P < 0.01 versus untreated controls. Data are representative of three independent experiments, each done in triplicate.

Chondrocytes are a major cell type in cartilage and produce different types of extracellular matrix. Aggrecan and type II collagen (Col 2) are major component of cartilage and chondrocyte markers [31]. The sources of inflammatory cytokines and proteolytic enzymes are not just the synovial cells but also the chondrocytes themselves, which contribute to cartilage destruction. We further investigated whether IL-20 upregulated the expression of inflammatory cytokines and proteolytic enzymes in chondrocytes. We used OACCs as the target cells for an *in vitro* assay. RTQ-PCR showed that IL-20 (200 ng/ml) induced IL-6, MCP-1, MMP-1, and MMP-13 expression in OACCs (Fig 3A). MMP activity assay also confirmed that IL-20 increased MMP activity in OACCs (Fig 3B).

### IL-20 downregulated TGF-β, BMP-2, aggrecan, and Col 2 in OACCs

Chondrogenesis, which is triggered by factors such as bone morphogenetic proteins (BMPs) [32] and transforming growth factor β (TGF-β) [33], leads to the expression of the master transcription factor SRY-box 9 (Sox9), which is essential for chondrocyte differentiation [34]. Sox9 controls the transcription of genes characteristic to the cartilage matrix, such as Col 2 and aggrecan, and it suppresses the subsequent formation of hypertrophic chondrocytes [11, 35, 36]. We therefore investigated whether IL-20 downregulated the expression of chondrogenic growth factor and chondrocytes marker. RTQ-PCR showed that IL-20 (200 ng/ml) downregulated TGF-β, BMP-2, Col 2, and aggrecan expression in OACCs (Fig 3C).

### IL-20’s effect on chondrogenic differentiation

Although IL-20 acts as a proinflammatory cytokine in OASFs and OACCs, the effect of IL-20 on chondrogenic differentiation has not been characterized. MSCs can differentiate into cells of mesenchymal lineage, such as chondrocytes. Therefore, we first estimated the effect of IL-20 on the chondrogenesis of mouse MSCs.

**Fig 4:**
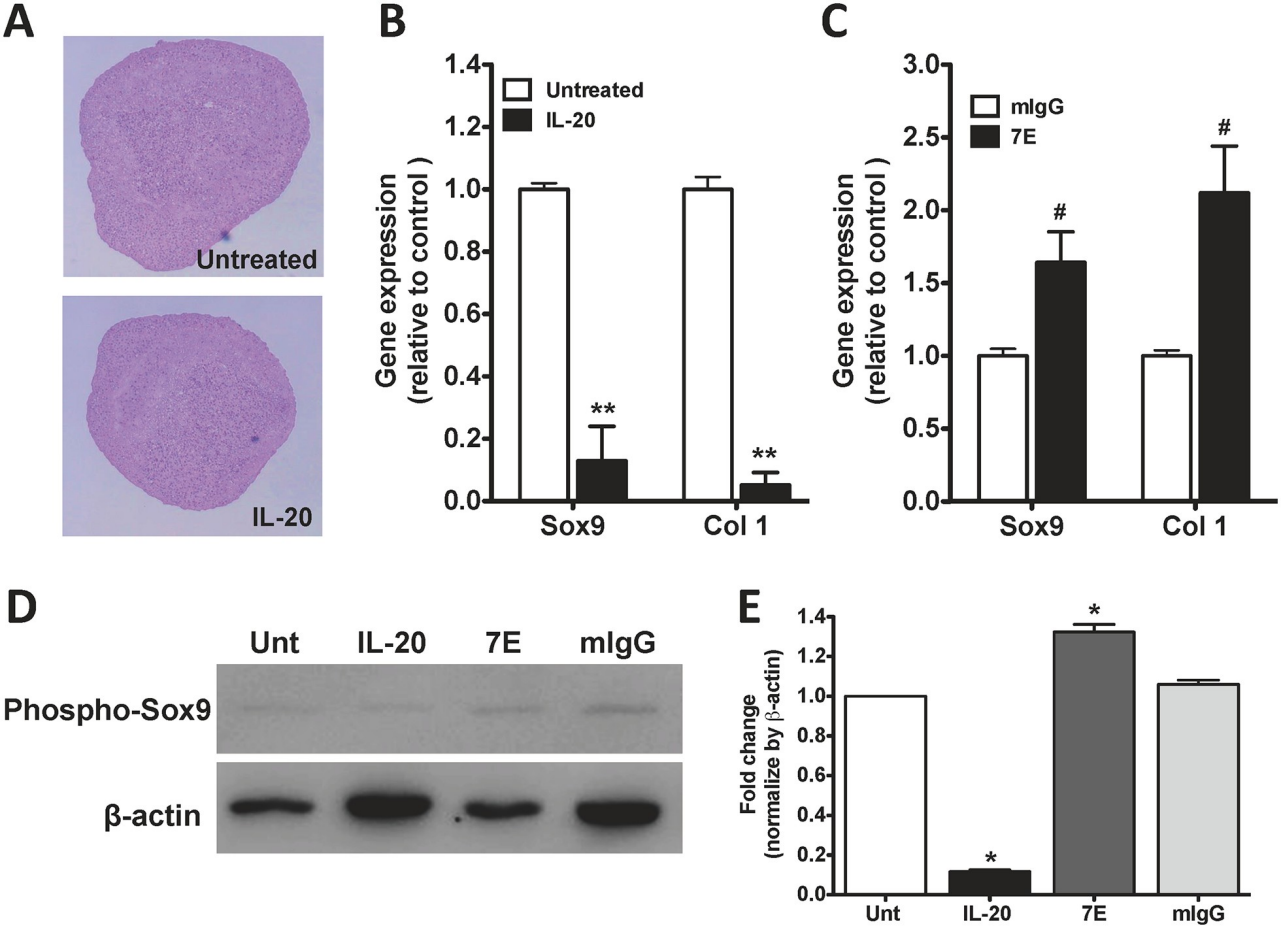
Correlation of IL-20 and chondrogenic differentiation. (A) Mouse mesenchymal stem cells (MSCs) were incubated with IL-20 (800 ng/ml) and cultured in chondrogenic medium. The cell pellets formed free-floating aggregates within the first 24 hrs. Aggregates cultured for 21 days were fixed and paraffin-embedded, and then sections were stained with Safranin O. (B-C) Mouse MSCs were incubated with IL-20 (800ng/ml), 7E (5 μg/ml), or mIgG (5 μg/ml) and cultured in chondrogenic medium for 7 days. Total RNAs were isolated from aggregates and analyzed using RTQ-PCR with primers specific for Sox9 and type 1 collagen (Col 1). The quantification analysis of mRNA was normalized; β-actin was the housekeeping gene. **P < 0.01 versus untreated controls. #P < 0.05 versus mIgG controls. Data are representative of three independent experiments, each done in triplicate. (D) Aggregates were cultured with IL-20 (800 ng/ml), 7E (5 μg/ml), or mIgG (5 μg/ml) for 7 days. Total cell lysates were analyzed using Western blotting with specific antibodies against phosphor-Sox9 and with β-actin. β-actin was used as an internal control. Data are representative of three independent experiments. (E) Bands were quantified using ImageJ software. *P < 0.05 versus untreated controls. Data are the means ± SD of three independent experiments.

Mouse MSCs were pellet-cultured in a chondrogenic induction medium containing TGF-β3 for 21 days, after which, Safranin O staining showed a marked increase in cartilage matrix molecule and proteoglycan expression; however, there were no significant changes in chondrogenic differentiation after IL-20 treatment compared with untreated controls (Fig 4A). To better characterize the effect of IL-20, the expression of chondrogenic marker genes were detected at three different stages: early (day 7), mid (day 14), and late (day 21). RTQ-PCR showed that Sox9 and type 1 collagen (Col 1) expression had been significantly inhibited in IL-20-treated (800 ng/ml) mouse MSCs during the early stage (Fig 4B). Sox9 and Col 1 expression in 7E-treated mouse MSCs had been upregulated during the early stage (Fig 4C). There were no significant changes in the mid or late stages after IL-20 treatment (data not shown). Additional mouse MSCs were pellet-cultured in a chondrogenic induction medium containing IL-20, 7E, or mIgG. The cell lysate was used for western blotting with an antibody specifically against phosphorylated Sox9. Analyses of Sox9 protein levels during the early stage of chondrogenic differentiation showed that phosphorylated Sox9 expression was lower in IL-20-treated MSCs but higher in 7E-treated than in mIgG-treated controls (Fig 4D and 4E).

### Arthritis severity and cartilage destruction in 7E-treated OA rats

**Fig 5:**
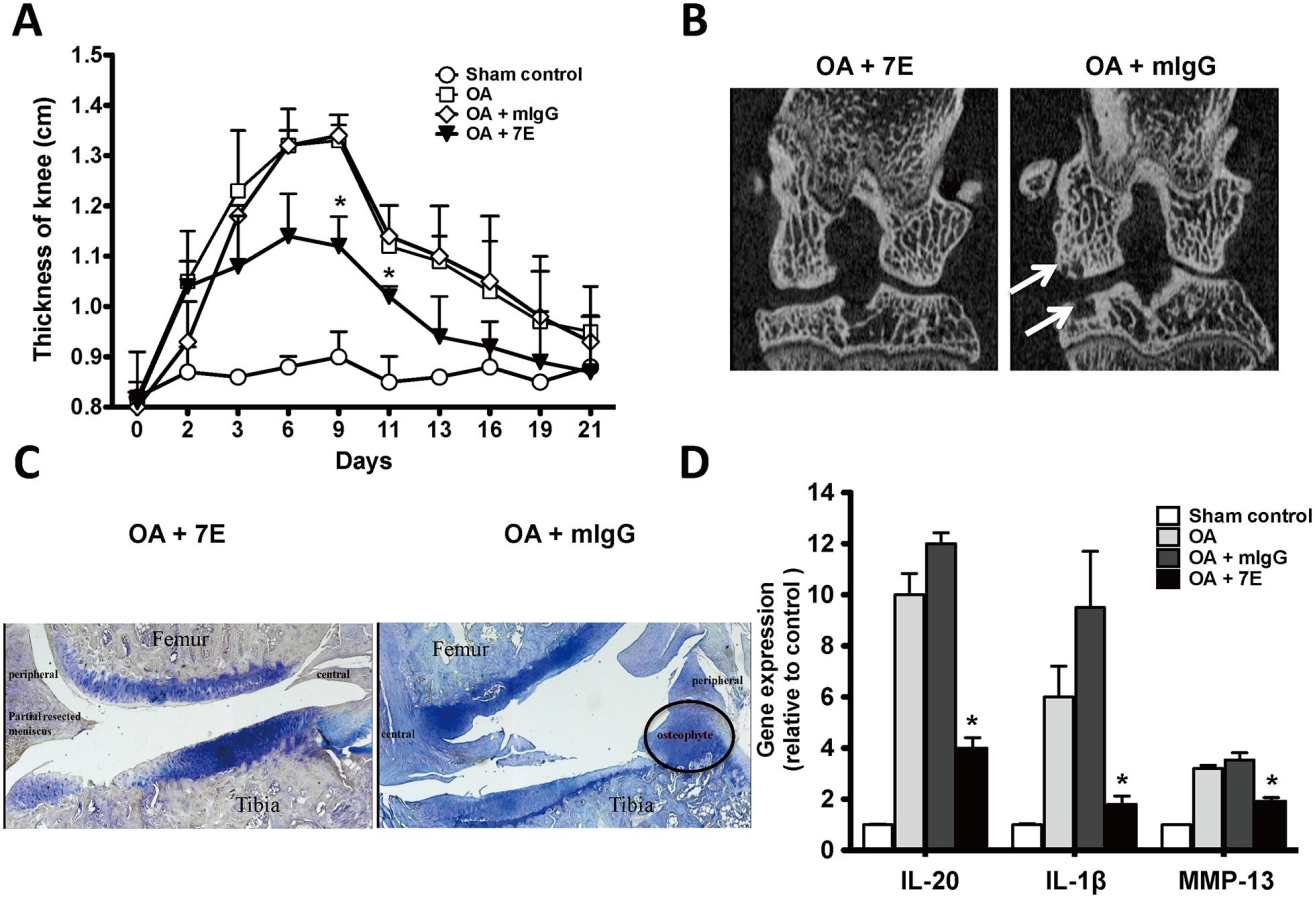
The effects of 7E on OA rats. Arthritis was surgically induced in 3 groups of rats (n = 5/group) on day 0. A fourth group of sham-operated rats were negative controls (n = 5). (A) Rats with OA were injected subcutaneously with 7E or mIgG (3 mg/kg) 3 times a week throughout the study. OA rats treated with phosphate buffered saline (PBS) were positive controls. Knee thickness as an indictor of disease activity was measured on the indicated days. *P < 0.05 versus mIgG-treated controls. Data are representative of three independent experiments. (B) On day 56, the rats were anesthetized, and radiographs were taken. Arrows indicate subchondral cysts. Representative radiographs are shown. (C) Histological sections of the knee joints of OA rats were stained using toluidine blue. Representative photos are shown. (D) On day 57, synovial tissue from rats was collected, mRNA was isolated and the IL-20, IL-1β, and MMP-13 transcripts were analyzed using RTQ-PCR with specific primers. The quantification analysis of mRNA was normalized; β-actin was the housekeeping gene. *P < 0.05 versus mIgG-treated controls. Data are representative of three independent experiments.

We previously reported [37] that 7E inhibited IL-20 *in vitro* and *in vivo*. We also analyzed all members of the IL-10 family (IL-10, -19, -20, -22, -24, and -26) to confirm the specificity of 7E using ELISA. Only IL-20 was recognized by 7E. To investigate whether 7E treatment attenuates OA severity, we generated surgery-induced OA in rats. The thickness of their knees (joint swelling) was significantly lower in 7E-treated (3 mg/kg) than in mIgG-treated OA rats (Fig 5A). Fifty-six days after the initial treatment, the severity of bone damage was radiologically examined in OA rats. Knee joints were more severely damaged in the mIgG-treated than in the 7E-treated OA rats. There was severe joint destruction and subchondral cyst formation in mIgG-treated OA rats but not in 7E-treated OA rats (Fig 5B). Toluidine blue staining also showed that less cartilage damage and osteophyte formation in 7E-treated OA rats (Fig 5C). To determine whether the attenuation of OA severity was correlated with inhibiting the expression of proinflammatory cytokines and MMPs, we isolated the knee synovial tissue from OA rats and used RTQ-PCR to analyze cytokine and MMP expression. Synovial expression of IL-20, IL-1β, and MMP-13 was significantly lower in 7E-treated rats than in the mIgG-treated rats (Fig 5D).

### Cartilage destruction in 7E- and MSC-treated OA mice

**Fig 6:**
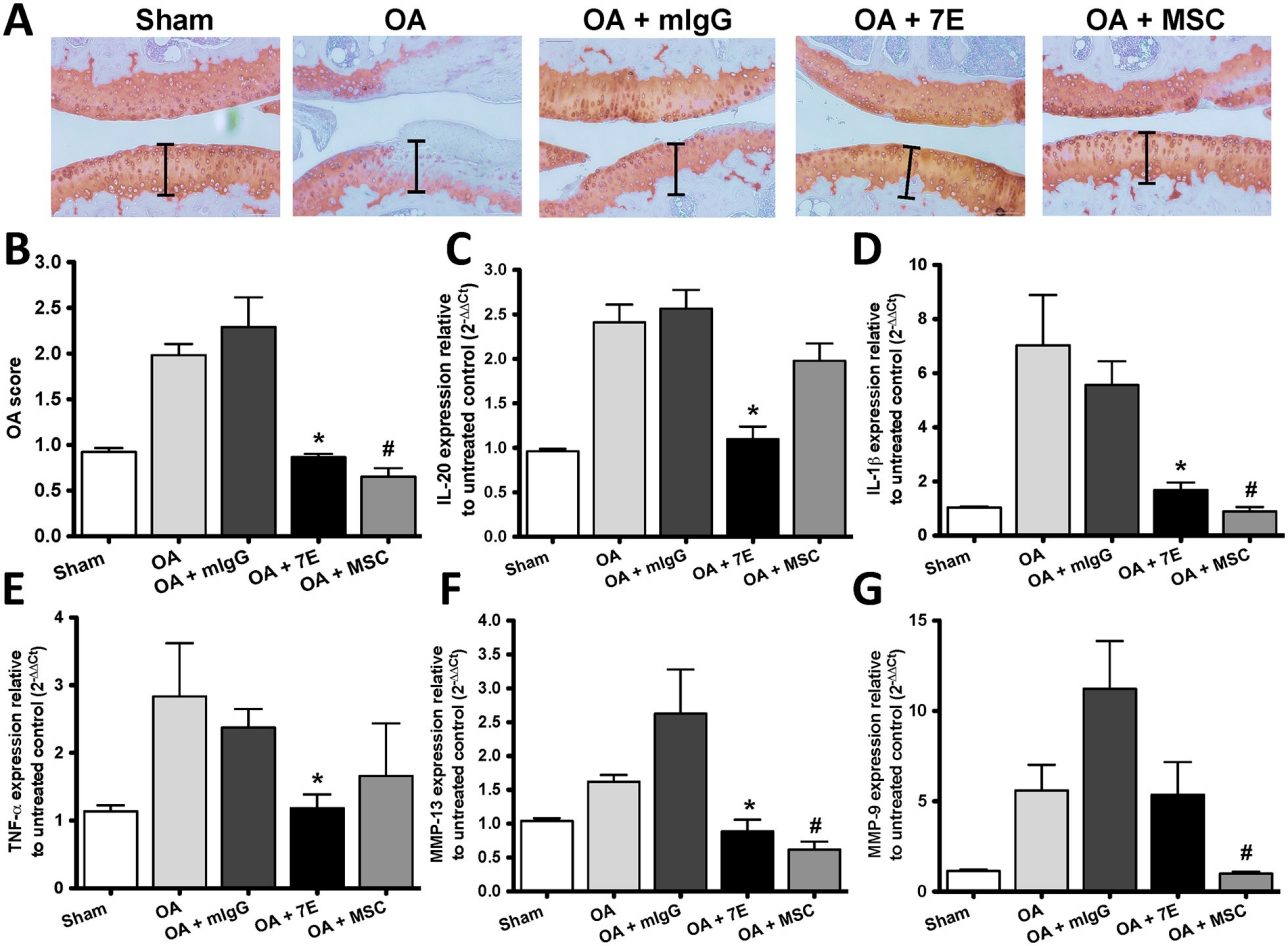
The effects of 7E and MSCs on OA mice. (A) Arthritis was surgically induced in 4 groups of mice (n = 5/group) on day 0. A fifth group of sham-operated mice were negative controls (n = 5). OA mice were given intra-articular injections of PBS, MSCs (5x10^5^/mouse/single injection), mIgG (2μg/mouse 3 times a week), or 7E (2μg/mouse 3 times a week) 14 days after surgery. Knee joint sections of the mice were stained with Safrainin O to analyze the effects of 7E and MSCs on destabilization of the medial meniscus (DMM) surgery-induced OA 42 days after treatment. Representative Safranin O stained images of cartilage sections are shown. (B) The severity of cartilage destruction was evaluated based on the scoring system using Safranin O images. Data are mean ± standard error of the mean (SEM) (n = 5 mice per group). *P < 0.05 versus mIgG-treated controls. (C-G) On day 56 after surgery, the knee joints from mice were collected, mRNA was isolated, and the IL-20, IL-1β, TNF-α, MMP-9, and MMP-13 transcripts were analyzed using RTQ-PCR with specific primers. The quantification analysis of mRNA was normalized; β-actin was the housekeeping gene. #P < 0.05 versus OA controls. *P < 0.05 versus mIgG-treated controls. Data are representative of three independent experiments.

Other studies [27, 29] showed that intra-articular injections of MSCs into OA knees improved knee joint function and reduced pain without causing adverse side effects, and reduced cartilage defects by regenerating hyaline-like articular cartilage. We therefore injected 7E (2μg/mouse 3 times a week) and 5x10^5^ mouse MSCs derived from bone marrow (single treatment) into the knee joints of OA mice 14 days after OA had been induced using DMM surgery. The knee joints were collected and stained with Safranin O 42 days after treatment. 7E- and MSC-treated mice have less cartilage damage than did mice in the Sham, untreated Control (OA), and mIgG-treated groups (Fig 6A). The OA score analysis also showed cartilage destruction was significantly inhibited in 7E- and MSC-treated mice (Fig 6B).

### Cytokine and MMP expression in the knee joints of 7E- and MSC-treated OA mice

The most relevant destructive effects of cytokines on the cartilage are mediated by MMPs. Among the members of the MMP family relevant roles are played by MMP-13, involved in the degradation of Col 2 in OA cartilage [38]. IL-1β stimulates the synthesis and activity of MMPs and other enzymes involved in cartilage destruction in OA [39]. To determine whether there was a correlation between the attenuation of OA severity and inhibiting cytokine and MMP expression, the knee joints of OA mice were analyzed. RTQ-PCR showed that IL-20, IL-1β, TNF-α, and MMP-13 expression was inhibited in 7E-treated OA mice (Fig 6C–6F), IL-1β, MMP-9, and MMP-13 expression was inhibited in MSC-treated OA mice (Fig 6D, 6F and 6G).

## Discussion

In the present study, we provide new evidence and show that IL-20 is involved in the pathogenesis of OA. IL-20 and its receptors were all expressed in the synovial fibroblasts and chondrocytes of OA patients and OA rats. IL-20 induced TNF-α, IL-1β, MMP-1, and MMP-13 expression by activating ERK-1/2 and JNK signals in OASFs. IL-20 not only upregulated MCP-1, IL-6, MMP-1, and MMP-13 expression, but also downregulated aggrecan, type 2 collagen, TGF-β, and BMP-2 expression in OACCs. *In vivo*, treatment with 7E significantly reduced the severity of arthritis and decreased cartilage destruction. Therefore, IL-20 directly affects local inflammation and cartilage damage, and indirectly affects these activities by inducing other mediators. We show that IL-20 acts as a proinflammatory cytokine in the microenvironment that affects the progression of OA.

We previously [14, 17] showed that synovial fibroblasts are the target cells of IL-20 in the pathogenesis of RA. In this study, we also showed that not only synovial fibroblasts, but also chondrocytes expressed both types of IL-20 receptor complexes in OA, which implies that chondrocytes are also one of the target cells of IL-20. In addition, we previously reported that IL-20 activated ERK-1/2 signaling in RASFs; therefore, in the present study, we examined the signal transduction pathway of IL-20 in OASFs and found that IL-20 activated ERK-1/2 and JNK signal transduction. This finding provides additional information about the molecular mechanism of IL-20 in the pathogenesis of OA. In addition, low oxygen tensions and hypoxia-inducible factor-1alpha (HIF-1α) are important factors in articular chondrocyte behaviour during cartilage homeostasis and osteoarthritis [40]. We previously [41] showed that IL-20 was upregulated in primary chondrocytes under hypoxia condition. Therefore, we speculated that hypoxic conditions might trigger IL-20 expression in the microenvironment of OA.

Cartilage destruction is another feature of OA and is associated with disease severity and poor functional outcome. In the progression of OA, IL-6, combined with IL-1β and oncostatin, upregulates MMP-1 and MMP-13 expression, and reduces Col 2 expression. MCP-1 induces MMP-1 and IL-6 expression and stimulates proteoglycan depletion [4]. We previously [42] reported that IL-20 combined with IL-1β contributed to the inflammation of herniated intervertebral discs by upregulating IL-8, MCP-1, and TNF-α. IL-1β and TNF-α were important contributors to the destruction of cartilage and bone in OA. In the present study, we found that IL-20 by itself induced the expression of several cytokines and MMPs in OASFs and OACCs, which suggested that IL-20 and other proinflammatory cytokines, such as TNF-α, IL-1β, and MCP-1, might synergistically to mediate the initial inflammatory response and lead to the progression of cartilage damage and bone destruction in the pathogenesis of OA.

IL-20 receptor mRNA was expressed at a low level in undifferentiated MSCs, but the level increased in MSCs incubated in chondrogenic culture medium (data not shown). This suggests that MSCs become sensitive to IL-20 stimulation in the early phase of chondrogenic development. Although IL-20 regulated Sox9 expression at the early stage of chondrogenic differentiation, there were no significant differences in chondrocyte differentiation in our *in vitro* culture system. The Sox9 transcription factor has essential roles in the chondrocyte differentiation pathway and is required for the expression of Sox5 and Sox6 [35]. However, IL-20 did not affect Sox5 or Sox6 (data not shown), which indicated that IL-20 has only a partial role in chondrogenic differentiation.

BMPs and TGF-β trigger the expression of Sox9, which is known to regulate the transcription of chondrocyte-specific genes such as Col 2 and aggrecan [32, 33]. IL-20 downregulated TGF-β, BMP-2, Col 2, and aggrecan expression in OACCs. In addition, IL-20 activated the phosphorylation of Sox9 in MSC-derived chondrocytes. These findings suggested that IL-20 not only directly affects Col 2 and aggrecan, but also indirectly affects these factors by regulating TGF-β and BMP-2.

The main goals of current OA therapy are controlling pain and improving joint function. Commonly prescribed OA medications include NSAIDs, other analgesics, locally administered corticosteroids, and viscosupplementation [4]. For many patients, the lack of disease-modifying OA drugs results in progressive cartilage damage that eventually necessitates surgical intervention. In our OA model, 7E treatment decreased cartilage damage and inhibited osteophyte formation. 7E potently reduced IL-1β and IL-20 expression in synovial tissue *in vivo*, which suggested that the active phase of IL-20 occurs earlier in the course of arthritic inflammation than do the active phases of IL-1β. Recent study [43] showed that treatment with NNC0109-0012 (anti-IL-20 mAb) was effective in patients with seropositive RA as early as week 1, with further improvements to week 12. NNC0109-0012 in combination with methotrexate, rapidly led to significant reductions in RA disease activity and patient-reported pain and global disease activity at 12 weeks compared to the placebo group. Therefore, we speculate that 7E might have a therapeutic potential for inhibiting early inflammation in OA development. In addition, the finding that 7E potently inhibited the expression of MMP-13 in synovial tissue *in vivo* also provided evidence that 7E might be a good therapeutic agent for cartilage damage.

Repairing articular cartilage is difficult because of the limited regenerative capacity of cartilage. One of the most promising biological approaches for articular cartilage repair is cell-based therapy using MSCs [44, 45]. Implanted MSCs has differentiated into chondrocyte-like cells and improved cartilage structure in several animal studies [46, 47]. In the present study, 7E and bone marrow-derived MSCs were equally efficacious in reducing the severity of arthritis by inhibiting the inflammatory response and by preventing cartilage damage in inflamed joints. Our RTQ-PCR analysis suggested that the protective effects of 7E and MSCs in OA mice might use different pathways. It is noteworthy that despite our surgery-induced OA model showing a critical role of inflammation in OA, the anti-cytokine approach has not yet proven significant improvement in OA symptoms and structure modification in clinical. OA is a much more complex disease, low-grade inflammation induced by the metabolic syndrome, innate immunity, aging, and post-trauma, which might cause different pathogenic phenotypes in OA patients [48, 49]. A more detailed delineation of the molecular mechanism required additional investigation.

IL-20 activates its type I (IL-20R1/IL-20R2) and type II (IL-22R1/IL-20R2) receptor complexes, which are also activated by IL-19 and IL-24. Other studies [50–52] have reported that IL-19 and IL-24 were involved in the pathogenesis of RA. Whether IL-19 and IL-24 are involved in the pathogenesis of OA awaits additional investigation.

In conclusion, our study shows that IL-20 acts as a proinflammatory cytokine in the local environment of OA development and progression. 7E reduced the severity of arthritis and cartilage destruction in OA rats and OA mice. Our findings provide evidence that IL-20 is a novel target and that 7E is a potential therapeutic agent for OA.

## Key messages

1. IL-20 acts as a proinflammatory cytokine in the local environment of OA development and progression.
2. Anti-IL-20 mAb reduced the severity of arthritis in murine models of surgery-induced OA.
3. IL-20 is a novel target and that anti-IL-20 mAb is a potential therapeutic agent for OA.

## Supporting information

S1 Fig Expression of IL-20 and its receptors in patients with OA. (DOCX)
