# Supplementary material for: Anti-IL-20 monoclonal antibody inhibited inflammation and protected against cartilage destruction in murine models of osteoarthritis
Source: PLoS One. 2017 Apr 20;12(4):e0175802. doi: 10.1371/journal.pone.0175802 (PMC5398531; doi:10.1371/journal.pone.0175802)

**S1 Fig. Expression of IL-20 and its receptors in patients with OA.** (A) IL-20 receptors (IL-20R1, IL-20R2, and IL-22R1) expression in osteoarthritis (OA) synovial tissue and cartilage was detected using immunohistochemical (IHC) staining with anti-IL-20R1, anti-IL-20R2, and anti-IL-22R1 monoclonal antibodies. Staining with mouse immunoglobin G1 (mIgG1) isotype as the primary antibody was the negative control for IL-20R1, IL-20R2, and IL-22R1. The reaction was detected using AEC chromogen stain (red), and the nuclei were counterstained with hematoxylin (blue). Magnification was 200×. (B) mRNA of the synovial tissue and cartilage from OA patients was isolated for RT-PCR analysis, using IL-20-, IL-20R1-, IL-20R2-, and IL-22R1-specific primers. GAPDH used as the housekeeping gene. All experiments were performed three times with similar results. Data are from a representative experiment.


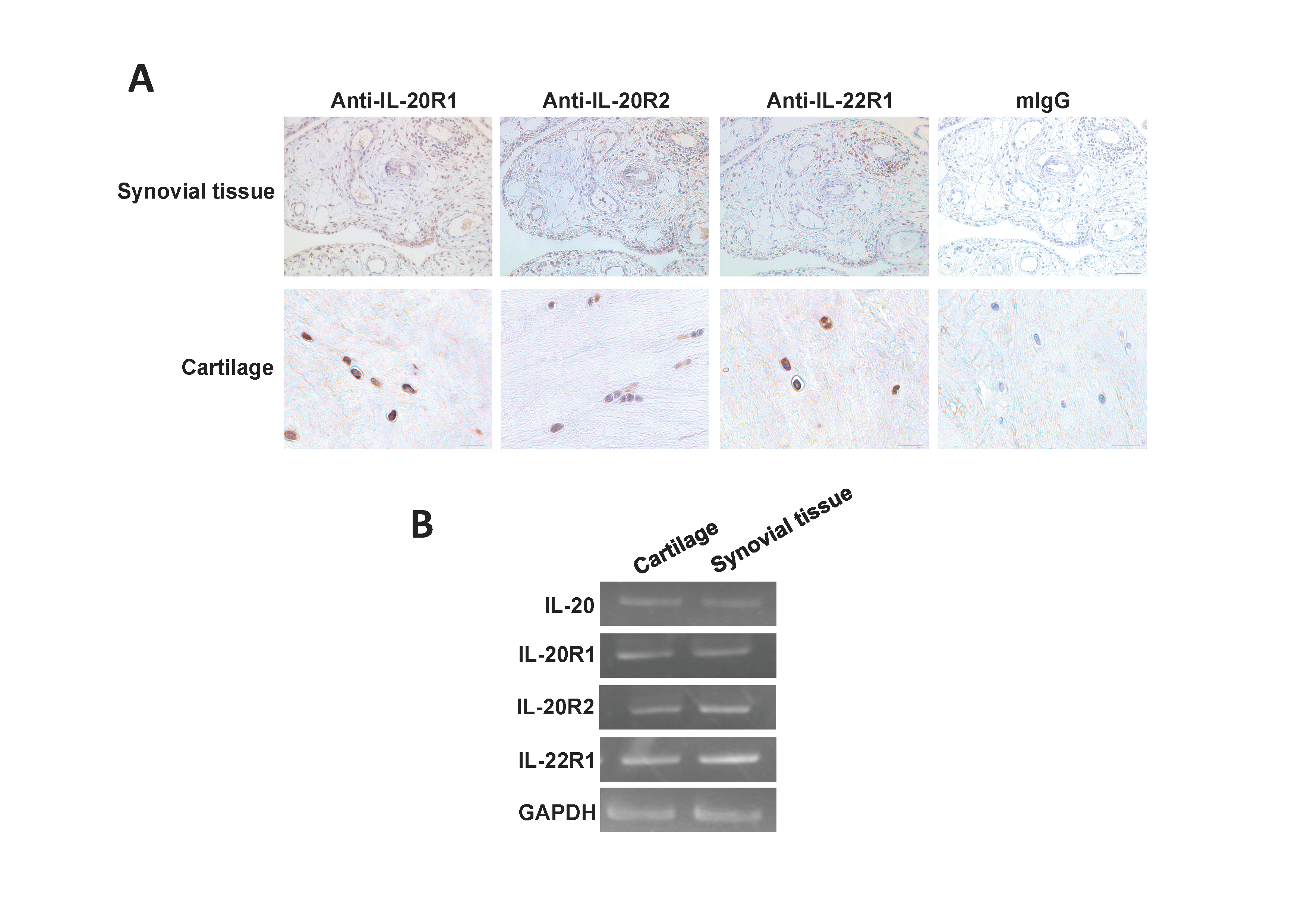

Supplement: S1 Fig — (DOCX) [file pone.0175802.s001.docx]
